# Monitoring Gastric Filling, Satiety and Gastric Emptying in a Patient with Gastric Balloon Using Functional Magnetic Resonance Imaging—A Feasibility Report

**DOI:** 10.4137/ccrep.s781

**Published:** 2008-05-22

**Authors:** Markus S. Juchems, Deniz Uyak, Andrea S. Ernst, Hans-Juergen Brambs

**Affiliations:** 1 Center for Diagnostic and Interventional Radiology, University Hospitals Ulm, Ulm, Germany.; 2 St.-Willehad Hospital, Department for Internal Medicine, Wilhelmshaven, Germany.

## Abstract

**Backround:**

Intragastric balloons are used for short term weight loss therapy in obese. It is possible to monitor the ballon with sonography, however this method is sometimes insufficient in obese patients. Therefore MRI seems to be a potential therapy-monitoring option.

**Purpose:**

In this feasibility report we want to demonstrate the potential use of functional MRI in monitoring gastric filling, patient satiation and gastric emptying in a obese patient who previously received intragastric balloon placement.

**Material and methods:**

We selected one patient (male, 178 cm, 127 kg, BMI = 40,5 kg/m^2^) who recently received a gastric balloon and visualized gastric motility in presence of the gastric balloon before and after food intake. Fast cross-sectional images in one breathhold spin echo or gradient echo sequences were aquired. Real-time gastric motion was performed with cine mode.

**Results:**

MRI offers perfect visualisation of gastric balloons in obese patients. Gastric filling and emptying can be monitored in correlation to patient satiety sensation. MRI can visualize the gastric balloon with degree of filling and possible leakages. Cine mode sequences demonstrate gastric motility and gastric wall peristalsis.

**Conclusion:**

MR is a valuable imaging alternative for patients with intragastric balloons

## Introduction

Intragastric balloons are available since the 1980s (1). They are designed for short-term (up to 6 month) weight loss therapy. The balloon device is deployed endoscopically. Once placed intragastrically, it is usually insufflated with saline water and discharged from the input device. However a recent publication by Mion et al. (2) demonstrated the potential value of air-filled balloons. There are currently two major indications for gastric balloon implantation. First for pre-surgery weight loss in severely obese patients and secondly for mid term weight loss in moderately obese patients along with a balanced diet. Several studies have been published about the effectiveness of gastric balloon placement. One of the largest trials has been carried out by Genco and co-workers (3) where 2515 patients with a mean BMI of 44,4 +/− 7,8 kg/m^2^ had balloons inserted since May 2000. After 6 month the average BMI decreased down to 35.4 +/− 11.8 kg/m^2^. Although this method is well investigated and the complication rate is considered to be low, there are possible complications ranging from patient intolerance (vomiting, discomfort, sensation of gastric reflux), partial or total balloon deflation, migration to severe complications like occlusion and perforation (4–7).

Factors that regulate food intake and satiation are complex and can not simply be cut down to bowel distension, as shown in a recent study by Oesch et al. (8). Nevertheless it seems that higher levels of balloon filling (600/800 ml) can at least decrease the degree of hunger.

Several studies have already been carried out—in paticular by the Zürich workgroup of Fried—that describe the possibility of visualizing gastrointestinal function using MRI. They showed that it is possible to monitor gastric emptying, accommodation, secretion motility and intragastral distribution of nutrition (9–11). MRI offers the possibility to acquire fast parallel cross-sectional images in one breathhold using spin echo (TSE, RARE) or gradient echo Sequences (True FISP). The sequences provide outstanding soft tissue contrast, thus enabling the investigator to differentiate gastric/bowel wall and gastrointestinal contents.

## Case Report

**Figure 1:**
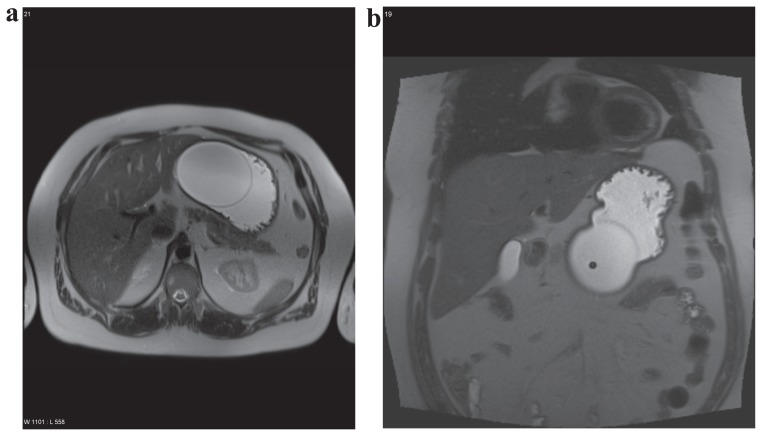
Axial (**a**) and coronar (**b**) T2w HASTE Sequence of the intragastral balloon and the stomach after the patient ingested 1L of tap water. The patient did not express satiety.

**Figure 2:**
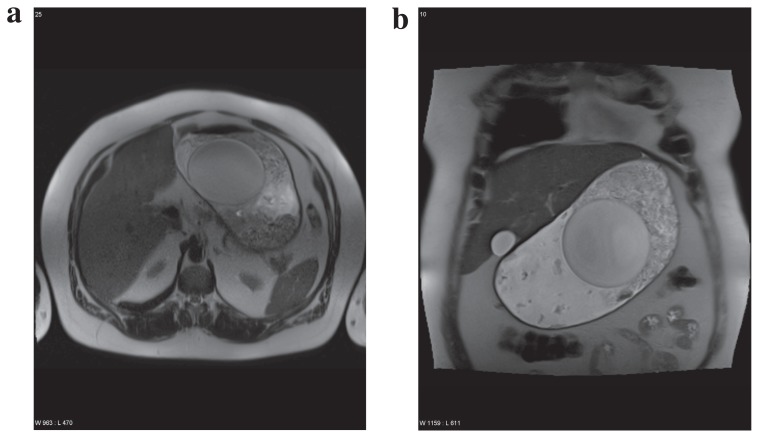
Axial (**a**) and coronar (**b**) T2w HASTE Sequence showing max. stomach distension after food intake of 500 mg noodles and a medium size salad. Furthermore 1500 ml of water were ingested. The patient expressed satiety.

In our particular case, we investigated a patient (178 cm, 127 kg, BMI = 40,5 kg/m^2^) who received an intragastral balloon (BIB® Intragastric Balloon, Allergan Inc., U.S.A.) filled with 500 ml saline water three month before the MRI examination. We carried out MRI examinations over a period of 9 h divided into 4 sub-series:

In the first test, we acquired images after filling the stomach with 1 liter of tap water (Fig. 1). Two hours later scans were undertaken without any additional water or food. After food intake (500 g noodles + a mid-size salad + 1500 ml tap water) we monitored gastric distension (Fig. 2) and gastric peristalsis. Finally, 4 hours after food intake we performed our last MRI series.

All scans were performed on a Simens Avanto 1,5T Scanner (Siemens Medical, Erlangen, Germany). We acquired coronar (2 mm; TE1.77, TR4.06) and axial (2 mm; TE1.77, TR4.06) gradient-echo sequences (trueFISP). Additionally coronar and axial T2 weighted sequenzes (axial: haste/5 mm/(TE104; TR1000); coronal: haste/5 mm/(TE92; TR1200); axial T2w BLADE/4 mm (TE99; TR4289)) spin-echo sequences as well as cine-mode sequences (T2w; TE 1.21 TR 43.35) and MIP reconstructions were acquired.

First, we were able to visualize the gastric balloon perfectly, given its high contrast in T2 weighted images caused by the saline water filling. With MRI it is—in contrast to plain radiography—easily possible to observe the position of the balloon in all three spatial planes for example if dislocation is suspected. Furthermore it is possible to assess the degree of filling if a leakage is suspected.

Several more interesting findings could be derived from our investigation. After drinking 1l of tap water before the first series, the patient did not express satiety. After two hours the stomach was completely emptied. Although a balloon was present, gastric emptying of liquids is performed similar to normal population.

In contrast after food intake we observed extreme gastric dilatation, the patient expressed satiety. The diaphragm was elevated about 5 cm on the left hand side. No tachycardia, vomiting or reflux was evident. Four hours after food intake, the stomach was completely emptied. Similar to emptying of liquids, emptying of nutrients is obviously not delayed compared to the normal population. Gastric wall peristalsis is not affected by the presence of a gastric balloon. Even more, the balloon is compressed during peristalsis thus giving it a more ellipsoid form factor.

As this is a feasibility report, more obese patients with gastric balloons need to be observed with MRI to draw a final conclusion of the value of this method. However MRI offers the possibility to monitor gastric filling and emptying in correlation to patient satiety. It might be possible in future to adapt balloon filling to the individual patient satiety sensation if a series of MRIs is performed prior to balloon implantation with different amounts of gastric filling and if the findings are correlated to patient bloating. As patients often describe hunger feeling adaption after a few weeks after a balloon has been implanted, MRI could probably help to visualize this subjective impression by showing a progressive ability of gastric wall distension. Future balloon design might then offer the possibility to adapt the balloon size by adding more volume.

## Conclusion

MRI offers perfect visualization of gastric balloons in obese patients. Gastric filling and emptying can be monitored in correlation to patient satiety sensation. Neither gastric emptying of liquids nor of food is delayed in the presence of a gastric balloon. If the stomach is filled after food intake, the balloon is deformed by gastric peristalsis.
